# Dual‐Triggered Near‐Infrared Persistent Luminescence Nanoprobe for Autofluorescence‐Free Imaging‐Guided Precise Therapy of Rheumatoid Arthritis

**DOI:** 10.1002/advs.202205320

**Published:** 2022-12-03

**Authors:** Ruoping Wang, Junpeng Shi, Qian Zhang, Qiang Peng, Xia Sun, Liang Song, Yun Zhang

**Affiliations:** ^1^ State Key Laboratory of Structural Chemistry Fujian Institute of Research on the Structure of Matter Chinese Academy of Sciences Fuzhou, Fujian China; ^2^ Xiamen Key Laboratory of Rare Earth Photoelectric Functional Materials Xiamen Institute of Rare Earth Materials, Haixi Institute Chinese Academy of Sciences Xiamen, Fujian 361021 China; ^3^ University of Chinese Academy of Sciences Beijing 100049 China; ^4^ Fujian Science and Technology Innovation Laboratory for Optoelectronic Information of China Fuzhou, Fujian 350108 China

**Keywords:** autofluorescence‐free imaging, persistent luminescence nanoprobe, rheumatoid arthritis, stimuli‐responsive

## Abstract

Rheumatoid arthritis (RA) is a common, chronic, and highly disabling autoimmune disease characterized by difficult treatment, long disease duration, and easy recurrence. The development and application of high‐sensitivity theranostic probes for RA that will facilitate precise monitoring of disease progression and enable effective treatment are currently hotspots in the field of RA theranostics. In this study, mZMI@HA, a dual‐triggered theranostics nanoprobe, is constructed based on near‐infrared persistent luminescence nanoparticles (NIR‐PLNPs) for precise RA treatment and therapeutic evaluation. This is the first reported use of high‐sensitivity autofluorescence‐free imaging based on NIR‐PLNPs for precise RA treatment and therapeutic evaluation. Compared with the NIR fluorescence imaging probe‐indocyanine green, the signal‐to‐background ratio of persistent luminescence (PersL) imaging is improved nearly 14‐fold. Using PersL imaging to guide photothermal therapy and controllable drug release through NIR/pH‐responsiveness, the progress of collagen‐induced RA is relieved. Additionally, the therapeutic evaluation of RA by PersL imaging is consistent with clinical micro‐computed tomography and histological analyses. This study demonstrates the potential of NIR‐PLNPs for high‐sensitivity imaging‐guided RA treatment, providing a new strategy for RA precise theranostics.

## Graphical abstract

Precise diagnosis and monitoring progression are of significant importance in the effective treatment and therapeutic evaluation of rheumatoid arthritis (RA). Here, a novel near‐infrared/pH dual‐triggered nanoprobe mZMI@HA, elicits excellent targeting abilities for the effective noninvasive diagnosis of RA with high sensitivity. Under the guidance of high‐sensitivity autofluorescence‐free imaging, the nanoprobe shows prominent therapeutic efficacy in ameliorating joint damage.

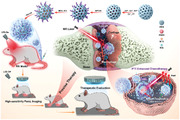

## 1 Introduction

Rheumatoid arthritis (RA) is a chronic and progressive autoimmune inflammatory disease characterized by persistent synovitis and progressive destruction of the cartilage and bone.^[^ ^1^ ^]^ As a progressive joint disease, if the symptoms are not detected and effectively controlled in a timely manner, RA can lead to joint injury and even permanent disability.^[^ ^2^ ^]^ Currently, the clinical diagnosis of RA is mainly established using radiography, magnetic resonance imaging, and computed tomography (CT).^[^ ^3^ ^]^ Although these imaging modalities provide opportunities for RA diagnosis and therapeutic evaluation, there are still deficiencies such as high cost, time consumption, ionization radiation, and complicated operations. Therefore, there is an urgent need to explore a highly sensitive, accurate, and secure diagnostic technique and formulate a reasonable and efficient treatment strategy to achieve precise RA therapy.

In recent years, owing to the development of nanotechnology, near‐infrared (NIR) nanoprobes have shown great potential in disease diagnosis and treatment.^[^ ^4^ ^]^ Owing to their advantages of high sensitivity, low cost, radiation‐free nature, and easy operation,^[^ ^5^ ^]^ NIR nanoprobes have become the best candidate for the diagnosis of RA. At present, researchers have simultaneously diagnosed and treated RA by properly designing a nanoprobe.^[^ ^6^ ^]^ Liu group developed iRGD peptide‐functionalized echogenic liposomes, which encapsulated methotrexate (MTX) and indocyanine green (ICG) using the thin film hydration method.^[^ ^7^ ^]^ This study confirmed that drug release under the guidance of NIR fluorescence imaging can greatly improve the therapeutic effect of RA. Zhang group designed a fluorescent organic nanoprobe, ONP‐CySe, for the non‐invasive diagnosis and treatment of RA. The therapeutic effect was monitored during treatment, which alleviated the degree of joint swelling and showed an excellent anti‐inflammatory effect.^[^ ^8^ ^]^ Kim group developed a novel anti‐RA nanoparticle, albumin‐ceria‐ICG, which could evaluate targeting ability and therapeutic effect under imaging guidance, effectively inhibiting the pathogenesis of RA.^[^ ^9^ ^]^ Nanotherapeutic probes based on fluorescent molecules have greatly advanced fluorescence imaging‐guided RA therapy. However, the currently reported fluorescent nanoprobes for RA theranostics rely on external excitation light irradiation in the diagnosis process, which undoubtedly causes background fluorescence interference and reduces imaging sensitivity. Therefore, the development of novel RA nanoprobes with high‐sensitivity diagnostic functions is of great significance for effectively monitoring and controlling the progression of RA, ultimately optimizing treatment strategies.

**Scheme 1:**
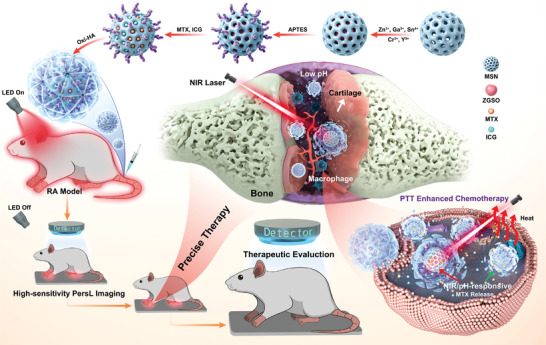
Schematic illustration of the fabrication process of the designed dual‐triggered theranostics nanoprobe and the high‐sensitivity autofluorescence‐free imaging‐guided RA treatment and therapeutic evaluation.

NIR persistent luminescence nanoparticles (NIR‐PLNPs) are unique optical nanoprobes that can emit NIR persistent luminescence (PersL) after excitation ceases.^[^ ^10^ ^]^ Such nanoprobes have unique characteristics for the separation of excitation and imaging,^[^ ^11^ ^]^ realizing autofluorescence‐free high‐sensitivity bioimaging,^[^ ^12^ ^]^ which makes them advantageous in the field of image‐guided precision therapy for RA. However, nano‐diagnostic probes for RA based on PLNPs have not yet been reported, resulting in a blank state of confirmatory research in RA precise diagnosis and treatment. Herein, we developed a dual (NIR and pH)‐triggered nanoprobe based on PLNPs for RA high‐sensitivity imaging‐guided treatment and therapeutic evaluation (**Scheme** **1**). First, novel renewable NIR‐emitting Zn_1.3_Ga_1.4_Sn_0.3_O_4_:Cr^3+^,Y^3+^ (ZGSO) PLNPs were synthesized via a facile mesoporous silica (MSN) template method, termed mZGSO. In addition, mZGSO was used as a vehicle to co‐load the clinical medicine‐MTX and the NIR‐responsive molecule‐ICG, this was termed mZMI. ICG endows mZMI with capabilities for both photothermal therapy (PTT) and NIR‐triggered drug controllable release. To improve the targeting ability of mZMI and prevent drug leakage, hyaluronic acid (HA) was selected as a gatekeeper and targeting ligand. In particular, HA was oxidized to obtain a dialdehyde structure and form oxidized hyaluronic acid (oxi‐HA), and mZMI was coated with oxi‐HA via Schiff base reactions. The custom‐designed nanoprobe (mZMI@HA) showed excellent precise drug‐controllable release capability via a dual NIR/pH‐triggered process on PersL imaging. In a collagen‐induced RA model, mZMI@HA achieved autofluorescence‐free imaging‐guided RA therapy with high sensitivity, specificity, and signal‐to‐background ratio (SBR). Thus, RA progression was almost completely suppressed. In addition, the therapeutic evaluation of mice with RA using PersL imaging was consistent with clinical test results. This study may offer valuable insights for exploiting PLNPs for RA precision medicine in the future.

## 2 Results and Discussion

### 2.1 Preparation and Characterization of mZGSO

**Figure 1:**
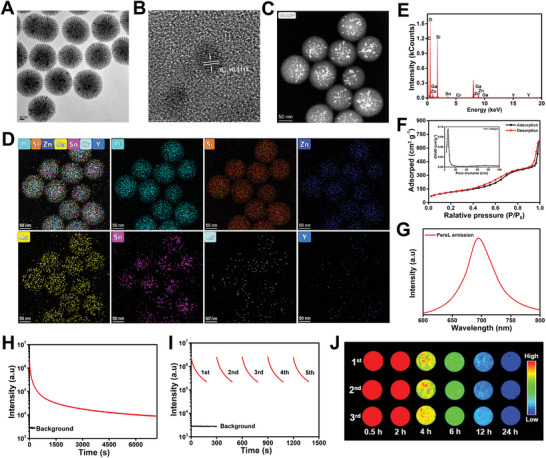
Characterization and optical properties of mZGSO. A) TEM image of mZGSO (scale bar: 20 nm). B) HAADF‐STEM image of mZGSO (scale bar: 5 nm). C,D) HAADF‐STEM EDS elemental mapping images of mZGSO (scale bar: 50 nm). E) EDS spectrum of mZGSO. F) N_2_ absorption–desorption isotherms of mZGSO (the inset is the corresponding pore size distribution). G) PersL emission spectrum of mZGSO after receiving 659‐nm LED light irradiation for 5 min. H) PersL decay curve of mZGSO after receiving 659‐nm LED light irradiation for 5 min (monitored at 700 nm). I) PersL decay curves of mZGSO after repeated irradiation by 659‐nm LED light (monitored at 700 nm). J) PersL images of mZGSO after receiving 659‐nm LED light irradiation for 5 min observed by charge‐coupled device (CCD) camera (exposure time: 60 s).

**Figure 2:**
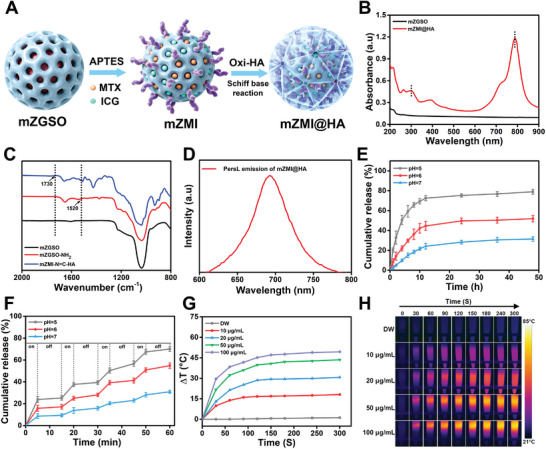
Surface modification and properties of mZMI@HA. A) Surface modification processes of mZMI@HA. B) UV–vis spectra of mZGSO and mZMI@HA. C) FTIR spectra. D) PersL emission spectrum of mZMI@HA upon 659‐nm LED light irradiation for 5 min. E) MTX controllable release of mZMI@HA in different pH condition without 808‐nm laser. F) pH/NIR‐triggered MTX controllable release of mZMI@HA. G,H) Temperature changes and IR thermal images of mZMI@HA at different concentrations under 808‐nm laser (1.0 W cm^−2^, 5 min).

mZGSO was synthesized via a facile MSN template method,^[^ ^13^ ^]^ which involved the synthesis of an MSN morphology‐controlled template,^[^ ^14^ ^]^ followed by the formation of the ZGSO phase inside the pores by ionic impregnation and calcination. As shown in **Figure** **1**A, the prepared mZGSO consisted of uniform, monodisperse, and spherical NPs with an average size of approximately 100 ± 5 nm. The hydrated particles were slightly larger (Figure S1, Supporting Information). High‐angle annular dark‐field scanning transmission electron microscopy (HAADF‐STEM) and energy‐dispersive X‐ray spectroscopy (EDS) elemental mapping were performed to verify the elemental composition and distribution. The HAADF‐STEM image of ZGSO showed a lattice fringe spacing of 0.3117 nm, which corresponded to the spacing of the (511) lattice planes (Figure 1B).^[^ ^15^ ^]^ The major elements Zn, Ga, Sn, O, Cr, and Y were well located in the porous structure of MSN (Figure 1C,D). EDS results also confirmed the presence of these elements (Figure 1E). Furthermore, the quantitative analyses of the metal elements in mZGSO were detected by inductively coupled plasma optical emission spectrometry (ICP‐OES). The results showed that the concentrations of Zn, Ga, Sn, Cr, and Y were 130.56, 145.976, 53.151, 0.373, and 0.4 parts per million (ppm), respectively (Figure S2, Supporting Information). In addition, the X‐ray powder diffraction (XRD) patterns indicated that all characteristic XRD peaks of mZGSO matched those of the ZnGa_2_O_4_ crystal (Figure S3, Supporting Information).^[^ ^16^ ^]^ All these results confirmed that mZGSO was the desired target product. Subsequently, the surface area, pore volume, and pore size of MSN and mZGSO were measured using nitrogen adsorption‐desorption isotherms. As shown in Figure 1F and Figure S4, Supporting Information, the hysteresis loop of the nitrogen adsorption‐desorption isotherms (typical type‐IV isotherms) revealed that MSN and mZGSO belong to the class of mesoporous materials.^[^ ^17^ ^]^ According to the Brunauer‐Emmett‐Teller and density functional theory methods, the surface areas of MSN and mZGSO were 774.005 and 414.986 m^2^ g^−1^, respectively, with pore volumes of 1.589 and 0.991 ccg^−1^, respectively. The mean pore size of mZGSO was 6.53 nm, indicating that it was an ideal candidate for drug loading. Next, the PersL properties of mZGSO were verified upon 659‐nm LED light irradiation, and a strong NIR PersL emission peak at 700 nm was observed (Figure 1G). Moreover, the NIR PersL of mZGSO exhibited a slow signal decay (**Figure** **2**H), and the PersL signal could be re‐excited by 659‐nm LED light repeatedly (Figure 1I). The same phenomenon was confirmed using a charge‐coupled device camera (Figure 1J). Such durable and renewable PersL of mZGSO ensured enduring intravital imaging, unrestricted by autofluorescence and luminescence duration.

### 2.2 Surface Modification, Drug Controllable Release, and Photothermal Property of mZMI@HA

The modification procedures for mZMI@HA are illustrated in Figure 2A. MTX and ICG were first loaded into amino‐modified mZGSO via the physical embedding method, and then mZMI was coated with oxi‐HA via Schiff base reactions.^[^ ^18^ ^]^ UV–vis absorption curves indicated the successful encapsulation of MTX and ICG in mZMI@HA (Figure 2B). DLS and zeta potential analyses also revealed successful modification of bare mZGSO (Figures S5 and S6, Supporting Information). Furthermore, FTIR spectra were used to evaluate the preparation of oxi‐HA and characterize mZMI@HA. There was an obvious new absorption peak (1730 cm^−1^) in the spectrum of oxi‐HA compared with prime HA (Figure S7, Supporting Information), owing to the absorption peak of the stretching vibration of the aldehyde group (—CHO), which confirmed the successful programming of oxi‐HA. For amino‐modified mZGSO, the typical stretching vibration absorption peak (1520 cm^−1^) of the amino group (—NH_2_) was observed and compared with that of mZGSO (Figure 2C). However, the characteristic absorption peaks of both —CHO and —NH_2_ disappeared for mZMI—N = C—HA (mZMI@HA) because of the Schiff base reaction between the —NH_2_ of mZGSO—NH_2_ and the —CHO of oxi‐HA, proving the successful preparation of mZMI@HA. Next, the stability of mZMI@HA was investigated. As shown in Figure S8, Supporting Information, after dispersing in three different solvents (Deionized Water, pH 7.4 PBS, 10% fetal bovine serum) at room temperature for 24 h, no significant aggregation in hydrodynamic size of mZMI@HA was discovered. These results indicated that mZMI@HA owned a high stability for further biological application. Subsequently, we analyzed the optical properties of mZMI@HA. Upon 659‐nm LED light irradiation, the same NIR PersL emission peak at 700 nm was observed for mZMI@HA (Figure 2D), and mZMI@HA also exhibited a slow signal decay like mZGSO (Figure S9, Supporting Information), laying the foundation for bioimaging.

The entrapment efficiency was verified by UV–vis spectrophotometry according to the absorption of ICG at 780 nm (Figure S10A, Supporting Information) and MTX at 303 nm (Figure S10B, Supporting Information), as calculated from the calibration curves of ICG (Figure S10C, Supporting Information) and MTX (Figure S10D, Supporting Information). To simultaneously achieve the ideal PersL signal and PTT of mZMI@HA, an optimal weight ratio (mZGSO:ICG = 4:1) with an entrapment efficiency of 99.8 ± 0.17% was employed for further MTX loading. As shown in Figure S11, Supporting Information, when the weight ratio between MTX and mZGSO was 1:2, the entrapment and loading efficiencies were optimal at 81.23 ± 0.2% and 31 ± 0.24%, respectively. Therefore, the final weight ratio of mZMI@HA (mZGSO:MTX:ICG = 4:2:1) was selected for further experiments.

Studies have shown that the synovial fluid pH in RA joints is lower than normal synovial fluid, which presents an acidulous environment. The reason for this may be due to the rapid proliferation and increased cellular metabolism of abundant infiltrated inflammatory cells in RA joints, lead to the local acidosis.^[^ ^19^ ^]^ Therefore, we simulated an acidic microenvironment to assess the pH‐triggered MTX release of mZMI@HA in vitro. As shown in Figure 2E, pH‐triggered MTX release without laser from mZMI@HA was investigated in phosphate buffers at different pH conditions (pH = 5, 6, 7). It could be clearly found that MTX release was pH‐dependent, the release rate of MTX gradually increased with the decrease of pH. The results revealed that mZMI@HA was able to realize pH‐responsive drug release, prompting the selective release of MTX in the RA environment. Next, NIR‐triggered MTX release of mZMI@HA was also tested. Under the alternate 808‐nm laser irradiation, the cumulative release amounts of MTX reached 70 ± 0.2% within 60 min (Figure 2F). Interestingly, the MTX release rate was laser‐dependent. When laser irradiation was received, a burst release occurred; otherwise, a slow release of MTX was observed which indicated that NIR demonstrably accelerated the drug release rate in a controllable mode. All results revealed that mZMI@HA had a good ability to control MTX release via NIR/pH‐responsive processes.

Considering the excellent NIR absorption of ICG, which endows mZMI@HA with PTT for RA, the photothermal conversion capability of mZMI@HA in deionized water (DW) was systematically investigated. As expected, the temperature of the aqueous‐dispersed mZMI@HA could be effectively increased under 808‐nm laser irradiation in a concentration‐dependent manner (Figure 2G). By increasing the concentration from 10 to 100 µg mL^−1^, the temperature changes of the solution increased by 49.4 °C upon 808‐nm laser irradiation in 5 min (1.0 W cm^−2^), which was sufficient to ablate inflammatory cells. In contrast, a negligible temperature increase was observed for DW under the same conditions. The temperature changes in the various solutions were also recorded by infrared (IR) thermal images (Figure 2H). For evaluating the conversion of photo energy into thermal energy, the photothermal conversion efficiency (*η*) of mZMI@HA was calculated to be 22.58% (Figure S12, Supporting Information). Furthermore, photothermal stability of mZMI@HA was also tested, a slightly reduction of temperature elevation was discovered when receiving irradiation with 808‐nm laser for four on‐and‐off cycles (Figure S13, Supporting Information). All results indicated that mZMI@HA was an excellent PTT platform.

### 2.3 Targeting Ability and Therapeutic Efficacy of mZMI@HA In Vitro

**Figure 3:**
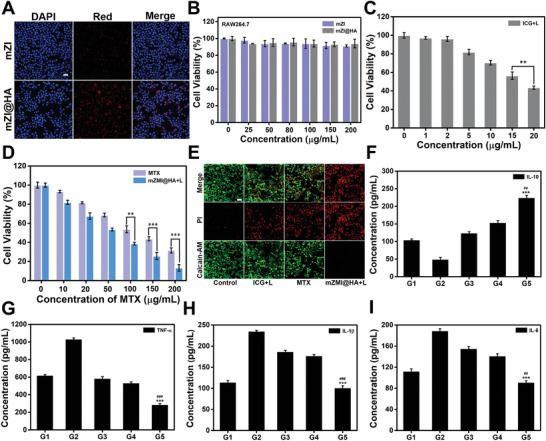
In vitro targeting ability and therapeutic effect of mZMI@HA. A) Cell images of LPS‐stimulated RAW264.7 cells treated with mZI or mZI@HA observed under confocal laser scanning microscopy (scale bar: 25 µm). B) Verification of cellular viability after different treatments of mZI or mZI@HA at various concentrations. C) In vitro cytotoxicity of ICG with laser (^**^p < 0.01). D) In vitro cytotoxicity of free MTX or mZMI@HA with laser (^***^p < 0.001, ^**^p < 0.01). E) Cell images of LPS‐stimulated RAW264.7 cells after different treatments and then being stained by calcein‐AM (green) and PI (red) observed under confocal laser scanning microscopy (scale bar: 25 µm). F–I) Inflammation level analyses results after different treatments (^***^p < 0.001, compared with control *^##^p* < 0.01, *^###^p* < 0.001 compared with MTX). G1: control (−), G2: model (+), G3: ICG with laser (+), G4: MTX (+), G5: mZMI@HA with laser (+). “‐” indicates that RAW264.7 cells received no LPS stimulation, and “+” indicates that RAW264.7 cells received LPS stimulation.

Activated macrophages show massive infiltration into RA synovial tissue, causing persistent chronic inflammatory responses.^[^ ^20^ ^]^ CD44, a potential target for RA therapy, is overexpressed on the membrane of activated macrophages and can specifically bind to HA.^[^ ^21^ ^]^ First, we indirectly verified that lipopolysaccharide (LPS)‐stimulated RAW264.7 cells were highly expressed CD44 receptor by HA blocking assay. As shown in Figure S14A,B, Supporting Information, compared with RAW264.7 cells with no treatment of LPS, LPS‐stimulated RAW264.7 cells presented more stronger cellular uptake behavior of mZI@HA. Meanwhile, the cellular uptake efficacy of mZI@HA was remarkably decreased in the LPS‐stimulated RAW264.7 cells with HA pre‐blocking. But there was no obvious luminescent intensity (red) of mZI@HA changed in the RAW264.7 cells with no treatment of LPS. Taken together, these results showed that the excellent cellular uptake behavior was due to the interaction between HA and CD44, which indirectly demonstrated the overexpression of CD44 receptor in the LPS‐stimulated RAW264.7 cells. The similar cellular uptake behavior of mZI@HA was also quantitatively analyzed by ICP‐OES (Figure S14C, Supporting Information). Next, to further verify whether the oxi‐HA‐coating of mZMI@HA could strengthen the cellular uptake efficacy by specific targeting, LPS‐stimulated RAW264.7 cells were incubated with mZI@HA or mZI. Compared with the control and mZI groups, a noticeable luminescent intensity (red) of mZI@HA was observed (**Figure** **3**A, Figure S15A, Supporting Information). In addition, the red mZI@HA signal weakened when the cells were pre‐blocking with HA, suggesting that oxi‐HA and CD44 have superior targeting ability and demonstrating an enhanced HA‐mediated cellular uptake behavior in LPS‐stimulated RAW264.7 cells. The significant difference in cellular uptake behavior of mZI@HA and mZI was shown in Figure S15B, Supporting Information.

The in vitro cytotoxicities of mZI@HA and mZI were evaluated in LPS‐stimulated RAW264.7 cells. After 24 h of incubation, no influence was found on cell viability, even at a concentration of 200 µg mL^−1^ (Figure 3B), indicating the good biocompatibility of the prepared nanoprobes. To guide drug release and PTT to achieve the highest therapeutic efficacy, we first validated the cellular uptake behaviors of mZI@HA at various time intervals. As shown in Figure S16A, Supporting Information, the cellular uptake behaviors of mZI@HA were time‐dependent, with the incubation time increased to 6 h, obvious red luminescent signals were detected under confocal laser scanning microscopy (CLSM). With the extension of incubation time, no significant difference in cellular uptake behaviors of mZI@HA was found (Figure S16B, Supporting Information). Further, the incubation time dependent cellular uptake behavior of mZI@HA was also quantitatively analyzed (Figure S14C, Supporting Information). These results indicated cellular uptake behaviors of mZI@HA basically reached saturation at 6 h. which was also considered as the best time point for cell therapy. Next, the therapeutic efficacy of mZMI@HA was assessed in LPS‐stimulated RAW264.7 cells using cell counting kit‐8 and live/dead cell staining analysis. As shown in Figure 3C,D, the cell viabilities were concentration‐dependent, and in the group treated with mZMI@HA with laser, the cell viability decreased to 13.4% at a concentration of 200 µg mL^−1^, compared with the cells treated with ICG with laser (43.5%) or free MTX (31.5%). This trend was attributed to the synergistic efficacy of PTT‐enhanced chemotherapy. Similar therapeutic effects were also observed in live/dead cell staining images (Figure 3E). To verify the anti‐inflammatory effect of mZMI@HA, we measured cytokine levels after the cells received different treatments; these had a high correlation with the severity of inflammation in the lesion.^[^ ^22^ ^]^ To construct an inflammatory environment in vitro, RAW264.7 cells were activated by LPS, and the activated macrophages released massive pro‐inflammatory cytokines with increased expression levels of tumor necrosis factor (TNF‐*α*), interleukin‐1*β* (IL‐1*β*), and interleukin‐6 (IL‐6), accompanied by a reduction in anti‐inflammatory cytokine interleukin‐10 (IL‐10) levels. As shown in Figure 3F, cells treated with mZMI@HA with laser exerted a superior anti‐inflammatory effect by increasing the expression of IL‐10. Meanwhile, overproduction of the pro‐inflammatory cytokines for TNF‐*α* (Figure 3G), IL‐1*β* (Figure 3H), and IL‐6 (Figure 3I) decreased dramatically. These findings demonstrate that the anti‐inflammatory efficacy of mZMI@HA with laser may present remarkable performance in RA therapy in vivo.

### 2.4 Targeting Ability and Therapeutic Efficacy of mZMI@HA In Vivo

**Figure 4:**
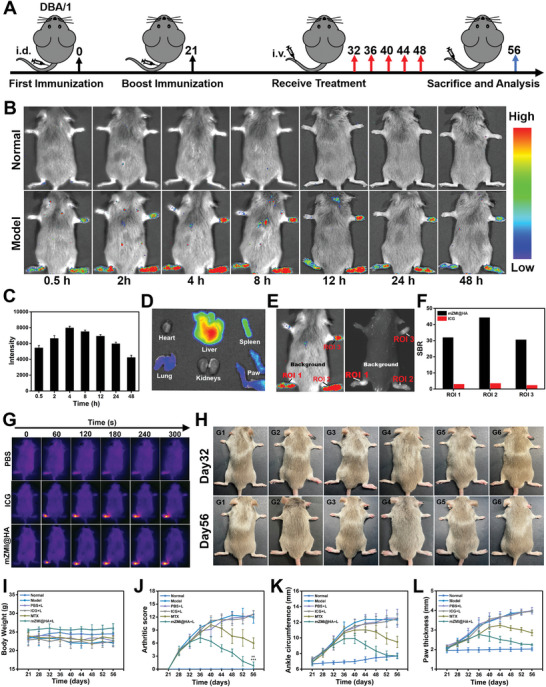
Targeting ability and therapeutic efficacy of mZMI@HA in vivo. A) RA model establishment and treatment time points. B) Representative PersL images of mice after treated mZMI@HA at different time points. C) Statistic analyses of PersL intensity derived from the RA joints at different time points (*n* = 3). D) Representative PersL images of the major organs derived from the typical RA mouse at the 48‐h time point. E) PersL imaging and fluorescence imaging at the 4‐h time point, the images were originated from one typical RA mouse (administration of mZMI@HA). F) SBR comparison between PersL imaging and fluorescence imaging derived from (E). Regions of interest (ROIs) were the PersL signal or fluorescence signal from the typical paws of mice (ROI 1 referred to the left hind paws, ROI 2 referred to the right hind paws, ROI 3 referred to the right forepaws). SBR was calculated by the PersL or fluorescence intensity of the paw versus the background. G) Infrared thermal images of RA mice at 4‐h time point following treatment with PBS, ICG, and mZMI@HA under 808‐nm laser (1.0 W cm^−2^, 5 min). H) Mice photographs on days 32 and 56. I–L) Recordings on body weight, arthritis score, average joint circumference, and paw thickness of mice during treatment (^***^p < 0.001, compared with normal mice *^##^p* < 0.01, *^###^p* < 0.001 compared with RA model of MTX). G1: normal mice, G2: RA model of PBS, G3: RA model of PBS with laser, G4: RA model of ICG with laser, G5: RA model of MTX, G6: RA model of mZMI@HA with laser.

An in vivo RA model was established using a collagen‐induced arthritis model in DBA/1 mice.^[^ ^23^ ^]^ The immunization reagent, intradermal (i.d.), was injected into the tail of the mice on days 0 and 21 (**Figure** **4**A). On day 32, the RA model was successfully established with symptoms of redness and swelling of the paws (Figure S17, Supporting Information). The targeting ability of mZMI@HA was evaluated using PersL imaging in mice with RA. As shown in Figure 4B, no luminescence intensity of the paws was detected in the control group (administration of mZMI@HA), whereas PersL images of the paws exhibited obvious signals in the RA model group (administration of mZMI@HA). As time passed, the PersL intensity increased in the group of RA mice injected with mZMI@HA, reaching maximum PersL intensity at 4 h post‐injection (Figure 4C). Additionally, the biodistribution of mZMI@HA was investigated ex vivo. At 48 h after intravenous injection of mZMI@HA, the main organs were harvested, and distinct PersL signals were observed in the liver, spleen, lung, and paws (Figure 4D), consistent with the results of in vivo imaging. To further validate the targeting ability of mZMI@HA for RA joint tissue, fluorescence imaging of ICG was performed, and a similar accumulation was observed (Figure S18, Supporting Information). Notably, autofluorescence‐free PersL imaging based on PLNPs showed high sensitivity and SBR. Especially at the 4 h time point, PersL images of the right hind paw displayed a significantly higher SBR of 44.4, compared with the ICG of 3.6 (Figure 4E,F). These results confirm the specific targeting ability of mZMI@HA in RA lesions. More importantly, the PersL imaging results indicated that high‐sensitivity imaging based on PLNPs was an ideal mode for precise RA diagnosis.

To evaluate the therapeutic efficacy of mZMI@HA, the RA mice were randomly divided into five groups (*n* = 5): PBS (G2), PBS with laser (G3), ICG with laser (G4), MTX (G5), and mZMI@HA with laser (G6). All mice received intravenous treatments once every four days. Five normal mice were intravenously injected with PBS as the control group (G1). Prior to assessment of the in vivo targeting ability of mZMI@HA, maximum accumulation was observed at 4 h post‐injection. Therefore, this time point was selected not only to guide PTT accompanied by precise MTX controllable release, but also to be considered as the optimal time point for RA diagnosis. As shown in Figure 4G, the paw temperature of RA mice treated with ICG or mZMI@HA quickly increased to 48 °C under 808‐nm laser irradiation, which was high enough to boost MTX release and efficaciously ablate inflammatory cells. At the end of treatment, RA symptoms almost disappeared in the mZMI@HA with laser group, and the mice treated with MTX were slightly relieved. In comparison, the treatments of ICG with laser, PBS, and PBS with laser failed to effectively inhibit inflammation (Figure 4H). During treatment, body weight fluctuated normally (Figure 4I). The arthritis score (Figure 4J, Figure S19, Supporting Information), average joint circumference (Figure 4K), and paw thickness (Figure 4L, Figure S20, Supporting Information) of the mice were also recorded to verify the effect of treatment more systematically. All results demonstrated the excellent therapeutic efficacy of mZMI@HA with laser.

### 2.5 Histopathology Evaluation and Anti‐Inflammatory Abilities In Vivo

**Figure 5:**
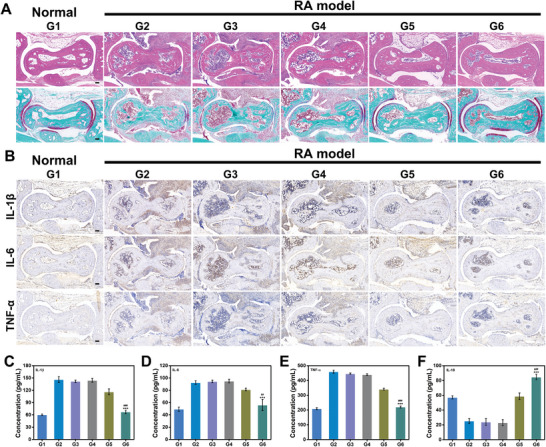
Histological analysis of joint synovium and inflammation levels. A) H&E and Safranin O staining of the ankle joint tissues in different groups. B) Immunohistochemical images of IL‐1*β*, IL‐6, and TNF‐*α* in ankle joint synovial tissues. C–F) Inflammation level analyses of serums (^***^p < 0.001, compared with normal mice *^##^p < 0.01*, *^###^p* < 0.001 compared with RA model of MTX). G1: normal mice, G2: RA model of PBS, G3: RA model of PBS with laser, G4: RA model of ICG with laser, G5: RA model of MTX, G6: RA model of mZMI@HA with laser.

Therapeutic efficacy of mZMI@HA was further evaluated using histological and immunohistochemical analyses. As shown in **Figure** **5**A, hematoxylin‐eosin (H&E) images of PBS, PBS with laser, and ICG with laser revealed severe synovium hyperplasia and multiple immune cell infiltrations. Mice treated with free MTX showed less extensive immune cell infiltration, while synovium hyperplasia was still obvious. The mZMI@HA group with laser irradiation showed less synovial hyperplasia and lower immune cell infiltration. Safranin O staining directly reflected the articular cartilage and bone structure under cartilage. Severe cartilage destruction was observed in the PBS, PBS with laser, and ICG with laser groups. In contrast, mice treated with mZMI@HA with laser demonstrated excellent cartilage protection abilities in alleviating synovial inflammation and reducing cartilage destruction, confirming the remarkable therapeutic effect of the treatment modality of PersL image‐guided precision therapy.

The anti‐inflammatory abilities of mZMI@HA were further assessed with immunohistochemistry. As shown in Figure 5B, the expression levels of pro‐inflammatory cytokines (TNF‐*α*, IL‐1*β*, and IL‐6) significantly increased in PBS, PBS with laser, and ICG with laser, while MTX showed a specific inhibitory effect on the secretion of pro‐inflammatory cytokines. The levels of pro‐inflammatory cytokines in the mZMI@HA group were significantly lower than those in the other groups. Furthermore, enzyme‐linked immunosorbent assay was used to investigate inflammatory regulation, which can directly reflect inflammation levels in vivo. In Figure 5C–E, the expression levels of IL‐1*β*, IL‐6, and TNF‐*α* were significantly increased in groups treated with PBS, PBS with laser, and ICG with laser, and compared with these three groups, the serum levels of the three aforementioned cytokines in the free MTX group were almost 1.25 (IL‐1*β*), 1.2 (IL‐6), and 1.3 (TNF‐*α*) times lower, respectively. More prominently, the secretion levels of IL‐1*β* (67.4 pg mL^−1^), IL‐6 (56.1 pg mL^−1^), and TNF‐*α* (220.6 pg mL^−1^) were significantly reduced compared with those in the other groups. In addition, mice treated with mZMI@HA exhibited a superior anti‐inflammatory effect by increasing the expression of IL‐10 (Figure 5F). All the above data revealed that our nanoprobe not only inhibited synovial hyperplasia and protected articular cartilage but also showed an excellent ability to regulate inflammation.

### 2.6 Therapeutic Evaluation of mZMI@HA In Vivo

**Figure 6:**
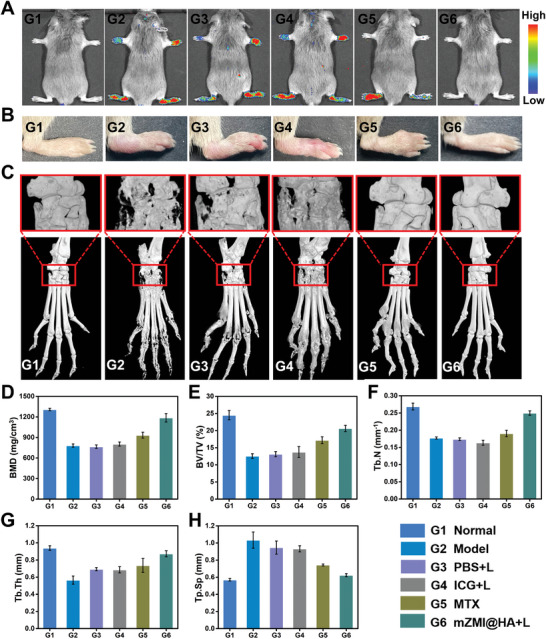
Validation of therapeutic evaluation after treatments. A) PersL images of mice after complete treatment. B,C) Representative pictures and micro‐CT images of the right hind paws on day 56. D,E) Quantitative micro‐CT analysis of BMD and BV/TV in ankle joints. F–H) Quantitative micro‐CT analyses of trabecular number (Tb.N), trabecular bone thickness (Tb.Th), and trabecular separation (Tb.Sp) in ankle joints.

As shown in **Figure** **6**A, mZMI@HA was further used for therapeutic evaluation, and no luminescence intensity was observed in the normal group, suggesting that mZMI@HA did not accumulate in normal mouse paws. Evident luminescence signals of all paws were observed in the PBS, PBS with laser, and free ICG with laser groups, indicating the excessive accumulation of mZMI@HA in the joints as RA symptoms worsened. Free MTX‐treated RA mice displayed a relatively low signal in the two paws with moderate symptoms. Interestingly, low luminescence intensity was detected in the mZMI@HA group, indicating that RA was significantly suppressed after treatment. These results demonstrate that high‐sensitivity PersL imaging of RA lesions using mZMI@HA has diagnostic potential for monitoring RA disease progression.

To verify the effectiveness and reliability of the PersL imaging results, hind paws in different groups were collected on day 56 for micro‐CT imaging (Figure 6B). As shown in Figure 6C, the inflamed ankle joints exhibited rough bone surfaces and serious bone erosion in the PBS, PBS with laser, and free ICG with laser groups, with a significant reduction in BMD and BV/TV, compared with the normal group (Figure 6D,E). Free MTX showed moderate efficacy in reducing bone erosion of the ankle joints, compared with the PBS group. Remarkably, treatment in the mZMI@HA with laser group showed smooth bone surfaces and had sufficient BMD, almost closer to the normal group, which effectively prevented bone destruction and loss. As shown in Figure 6F–H, the trabecular parameters also verified that treatment of mZMI@HA with laser was effective in increasing the trabecular number and trabecular bone thickness while decreasing trabecular separation. Similar results were found in the analysis of forepaws (Figure S21, Supporting Information). These results confirmed that this treatment modality could effectively ameliorate bone damage and simultaneously confirm the effectiveness and reliability of PersL imaging based on mZMI@HA.

### 2.7 Biodistribution and Biocompatibility Evaluation of mZMI@HA

The in vivo biodistribution of mZMI@HA in normal DBA/1 mice was assessed by ex vivo PersL imaging. Major organs (heart, liver, spleen, lungs, kidneys) were collected from the mice at different time points after intravenous administration of mZMI@HA. As shown in Figure S22, Supporting Information, the ex vivo PersL imaging showed that mZMI@HA was mainly taken up by the reticuloendothelial system (RES). The PersL signals reached maximum at 24‐h time point in liver, lung and spleen. Subsequently, mZMI@HA was gradually metabolized as the elapse of time. Next, we did the biocompatibility evaluation of mZMI@HA. First, a hemolysis test was used to evaluate the biocompatibility of mZMI@HA in vitro. The hemolysis rate of erythrocytes in DW reached 100%; however, mZMI@HA showed a hemolysis rate lower than 2.7% at a concentration of 800 µg mL^−1^ (Figure S23, Supporting Information) when incubated with whole blood for 4 h. Moreover, at the end of treatment, we collected major organs from all mice. Compared with normal mice, H&E images of major organs (heart, liver, spleen, lung, and kidney) demonstrated no obvious histological abnormalities or lesions following mZMI@HA administration, suggesting the excellent biocompatibility of mZMI@HA in vivo (Figure S24, Supporting Information). Finally, liver and kidney functions in the mice were assessed using blood biochemical tests. As shown in Figure S25, Supporting Information, there were no obvious changes in the liver function indices of alkaline phosphatase, alanine aminotransferase, and aspartate aminotransferase, as well as kidney function indices of blood urea nitrogen and creatinine.

## 3 Conclusion

A NIR/pH dual‐triggered nanoprobe (mZMI@HA) based on PLNP was established, that assisted with autofluorescence‐free NIR PersL imaging for RA imaging‐guided treatment and therapeutic evaluation. The high‐sensitivity PersL imaging mode allowed mZMI@HA to guide precise PTT and control drug release. The mZMI@HA treatment demonstrated excellent photostability and biocompatibility. Importantly, mZMI@HA showed excellent targeting ability in RA lesions 4 h post‐injection. Under the guidance of high‐sensitivity autofluorescence‐free PersL imaging, mZMI@HA presented a remarkable therapeutic effect on RA in inhibiting synovial hyperplasia and cartilage destruction, as well as regulating RA inflammation. Moreover, mZMI@HA had an excellent ability to accurately evaluate the presence of RA after treatment. All diagnostic results based on PersL imaging were validated by clinical methods using micro‐CT imaging and histological analysis, and similar results were obtained, suggesting the diagnostic potential for monitoring RA disease progression. In summary, this strategy indicates that the development of therapeutic nanoplatforms based on PLNPs is promising for RA precision medicine.

## 4 Experimental Section

### Materials

Zinc acetate dihydrate (Zn(CH_3_COO)_2_·2H_2_O, 99.99%), gallium nitrate (Ga(NO_3_)_3_·*x*H_2_O, 99.99%), yttrium (III) nitrate tetrahydrate (Y(NO_3_)_3_·4H_2_O, 99.99%), trimethylammonium chloride (CTAC), tetraethyl orthosilicate (TEOS), and triethanolamine (TEA) were purchased from Sigma‐Aldrich. Chromic acetate (Cr(CH_3_COO)_3_, 99.99%), tin (IV) chloride pentahydrate (SnCl_4_·5H_2_O, 99.9%), and MTX were purchased from Aladdin. Hyaluronic acid (97%, 40–100 KDa) was obtained from Macklin. ICG was obtained from Shanghai Sangon Biotech Co., Ltd., and 4,6‐diamino‐2‐phenylindole (DAPI) was acquired from Beyotime Biotechnology. Calcein AM‐PI dye was obtained from Thermo Fisher Scientific. Cell Counting kit‐8 (CCK‐8) and all cell culture supplies were obtained from ZetaLife. Cytokine detection kits for IL‐6, TNF‐*α*, IL‐10, and IL‐1*β* were purchased from Proteintech Group, Inc. Immunization‐grade bovine type II collagen lyophilized powder (10 mg), incomplete Freund's Adjust (5 mL), and complete Freund's Adjust (5 mg mL^−1^) were purchased from Chondrex.

### Characterization

Transmission electron microscopy images were obtained using a Hitachi H‐7650 system (Hitachi, Japan), and the nanoparticle size and zeta potential were determined using dynamic laser scattering (Brookhaven, USA). HAADF‐STEM and energy‐dispersive spectroscopy (EDS) elemental mapping were performed using an FEI Talos F200s transmission electron microscope (Thermo Fisher, USA) equipped with an energy‐dispersive X‐ray spectrometer (EDS). XRD patterns were measured using a Miniflex 600 X‐ray diffractometer (Rigaku, Japan). Cell images were observed under CLSM (Nikon, Japan). Quantitative analyses of metal elements were detected by ICP‐OES (Horiba Jobin Yvon S.A.S, France). Optical properties were recorded on an FLS920 spectrometer (Edinburgh, UK). UV–vis absorption spectra were obtained using a Cary 5000 spectrophotometer (Agilent, USA). Fourier‐transform infrared spectra were obtained using a Nicolet iS50 spectrometer (Thermo Fisher, USA). Bone parameters were analyzed using high‐resolution X‐ray micro‐CT (Bruker, Belgium).

### Cell Lines and Animals

All cell lines were acquired from the American Type Culture Collection. Male DBA/1 mice (7‐8 weeks old) were purchased from Gempharmatech Co., Ltd. All animal experiments were conducted in accordance with the protocols of the National Regulation of China for the Care and Use of Laboratory Animals. All animal experiments were approved by the animal ethics committee of Xiamen University (XMULAC20180037).

### Synthesis of Mesoporous Silica

The MSN were synthesized using a biphasic stratification method. Briefly, TEA (0.18 g) and 24 mL of CTAC aqueous solution (25 wt% in ultrapure water) were added to ultrapure water (36 mL), separately. After stirring for 1 h in an oil bath at 60 °C until no air bubbles were found in the reaction vial, TEOS (4 mL) mixed with cyclohexane (16 mL) was gently added to the mixture. Subsequently, the reaction was kept under magnetic stirring at 60 °C for 18 h. The final product was centrifuged at 11 000 rpm for 15 min and then washed with a mixture of ultrapure water and ethanol. To remove the CTAC template, the dried sample was annealed at 550 °C (2 °C min^−1^) for 5 h for further use.

### Synthesis of mZGSO

mZGSO was synthesized using the facile MSN template method. The precursor solution was formulated in advance with a final volume of 2.5 mL; it contained Zn(CH_3_COO)_2_·2H_2_O (0.428 g), Ga(NO_3_)_3_·*x*H_2_O (0.537 g), SnCl_4_ 5H_2_O (0.0 1577 g), Cr(CH_3_COO)_3_ (0.5 m, 15 µL), and Y(NO_3_)_3_·4H_2_O (0.01 m, 450 µL). Next, the precursor solution (500 µL) was mixed with MSN (100 mg) and evaporated in a vacuum oven at 60 °C overnight. Finally, the dry product was annealed at 850 °C for 3 h, and after cooling down to room temperature, mZGSO was obtained. For quantitative analyses the concentrations of Zn, Ga, Sn, Cr, Y metal elements in mZGSO, 5 mg of mZGSO was first treated with NaOH (2 m, 5 mL) for 24 h. After that, the mixed solution (HCl:HNO_3_ = 3:1, 5 mL) was added in the above solution for processing another 24 h. Then the above solution was volatilized by high temperature (160 °C) for the final volume of about 200 µL. Subsequently, the final solution was determined to 10 mL for further detection by ICP‐OES.

### Preparation of mZMI@HA

For the amination of mZGSO, mZGSO (200 mg) was ultrasonically dispersed in dimethylformamide (80 mL). Next, (3‐aminopropyl) triethoxysilane (500 µL) was added dropwise to the above solution and reacted at 60 °C for 24 h. Subsequently, mZGSO (mZGSO‐NH_2_) was obtained after centrifugation and several washes. For drug loading, 20 mg of mZGSO‐NH_2_ was mixed with MTX (10 mg) and ICG (5 mg) and stirred (800 rpm) at room temperature in the dark for 24 h after 30 min of ultrasound. Then, the mZMI was acquired after precipitating and washing three times with ultrapure water.

Oxi‐HA was prepared as described in a previous report. First, pure HA (400 mg) was dissolved in 40 mL of ultrapure water, and NaIO_4_ (320 mg) was dissolved in 12 mL of ultrapure water. Under magnetic stirring, NaIO_4_ solution was quickly added to the HA solution for 24 h in the dark. Ethylene glycol (5 mL) was then added to remove excess NaIO_4_, and the reaction was allowed to proceed for 30 min. Pure oxi‐HA was obtained for further use by dialysis and freeze‐drying.

mZMI was coated with oxi‐HA via Schiff base reactions. Briefly, mZMI (10 mg) was dispersed into 3 mL of MES buffer (0.01 m, pH = 7). After a brief ultrasound, mZMI solution was added tardily into the oxi‐HA solution, which was reacted in the dark for 24 h. Ultimately, mZMI@HA was collected by freeze‐drying after purification via centrifugation and washing. A UV–vis spectrophotometer was employed to analyze drug loading and entrapment efficiency, which were calculated using the following equations:

(1) $$\begin{array}{ccc} \mathrm{DL} & = & \left(\mathrm{Quality}\;\mathrm{of}\;\mathrm{loaded}\;\mathrm{drug}/\mathrm{Quality}\;\mathrm{of}\;\mathrm{drug}-\mathrm{loaded}\;\mathrm{nanoprobe}\right)\\ & & \times \;100\% \end{array}$$

(2) $$\mathrm{EE}=\left(\mathrm{Quality}\;\mathrm{of}\;\mathrm{loaded}\;\mathrm{drug}/\mathrm{Total}\;\mathrm{dosage}\right)\times 100\%$$

The stability of mZMI@HA was examined by monitoring the dimensional changes by DLS. Briefly, mZMI@HA (1 mg mL^−1^, 2 mL) was well dispersed in different solvents (Deionized Water, pH 7.4 PBS, 10% fetal bovine serum) at room temperature for a certain time (0 or 24 h), followed by measuring the change of their sizes by DLS.

### Photothermal Property and Methotrexate Controllable Release of mZMI@HA In Vitro

For photothermal properties, mZMI@HA was dispersed in deionized water at various concentrations (0, 10, 20, 50, and 100 µg mL^−1^) and irradiated with an 808‐nm laser (1 W cm^−2^, 5 min). Temperature changes were recorded using an infrared thermal imager. Photothermal stability performance of mZMI@HA (40 µg mL^−1^) was detected by 808‐nm irradiation (1.0 W cm^−2^, 5 min) for four on‐and‐off cycles. To evaluate the photothermal conversion efficiency of mZMI@HA, 0.8 mL of mZMI@HA at a concentration of 40 µg mL^−1^ was received irradiation under an 808‐nm laser (1 W cm^−2^, 5 min). The photothermal conversion efficiency was calculated by the reported equations below:

(3) $$\mathrm{Photothernal}\;\mathrm{conversion}\;\mathrm{efficiency}\;\mu =\frac{\mathrm{hs}\left(T_{\mathrm{Max}}-T_{\mathrm{Surr}}\right)-Q_{\mathrm{Dis}}}{I{\left(1-10\right)}^{-A_{808}}}$$

*hs* can be calculated by the ratio of the linear relationship of time *t* versus −ln*θ*. *T*_max_ and *T*_Surr_ refer to the maximum steady state temperature and the environmental temperature, respectively. *Q*_Dis_ represents heat dissipated from the laser mediated by the DW and container. *I* is the optical power and *A* is the absorbance at 808 nm of mZMI@HA.

Drug release capacities were determined by precipitation. For pH‐responsive drug release, mZMI@HA (2 mg) was dispersed in different pH solutions (pH = 5, 6, and 7) in a volume of 4 mL, respectively. At specific time points, each solution was centrifuged, and the supernatant was collected for UV–vis analysis, accompanied by replacement with fresh solution. The amount of drug released was calculated using the standard curve of MTX. For NIR‐responsive drug release, in addition to adding an 808‐nm laser every 10 min, the remaining experimental steps were consistent with those mentioned above.

### Cell Imaging Analyses

For validation of the CD44 overexpression on RAW264.7 cells, the cells were seeded in 6‐well plates for 6 h. Then the groups of LPS‐stimulated RAW264.7 cells were treated with LPS (100 ng mL^−1^) for another 24 h. For HA blocking, LPS‐stimulated RAW264.7 cells were pre‐incubated with HA (10 µg mL^−1^) for 8 h. Next, a fresh medium containing 50 µg mL^−1^ of mZI@HA was incubated with the cells in different groups for 6 h. For cell images analyses, the cells were fixated with 4% paraformaldehyde, and then stained with 4, 6‐diamino‐2‐phenyl indole (DAPI) for further observation via a confocal laser microscope under excitation at 405 nm (emission ranging from 425 to 475 nm) for DAPI and 561 nm (emission ranging from 663 to738 nm) for mZI@HA. For quantitative analyses of cellular uptake behaviors of mZI@HA, the cells were collected and processed according to the above method for ICP‐OES analysis. Targeting capability and incubation time dependent cellular uptake behaviors of mZI@HA were implemented via the similar steps as the above mentioned.

### In Vitro Cell Experiments

CCK‐8 assay was employed for cytotoxicity analyses, and RAW264.7 cells activated by LPS (100 ng mL^−1^) were incubated with different concentrations of mZI@HA or mZI (0, 25, 50, 80, 100, 150, and 200 µg mL^−1^). After incubation for 24 h, the cells were washed with PBS and treated with serum‐free medium (100 µL) containing CCK‐8 solution (10 µL). The absorbance of the cells was determined at 450 nm using a microplate reader. To evaluate the therapeutic effect in vitro, RAW264.7 cells activated by LPS (100 ng mL^−1^) were seeded in 96‐well plates and cultured for 24 h. Then, the cells were treated with mZMI@HA containing various concentrations of MTX (0, 10, 20, 50, 100, 150, and 200 µg mL^−1^), and the ICG with laser group received different concentrations of ICG (0, 1, 2, 5, 10, 15, and 20 µg mL^−1^). After incubation for 6 h, the old culture medium was discarded, and the cells were tenderly washed several times. The culture wells were replenished with fresh culture media (200 µL), and the mZMI@HA and ICG groups received an 808‐nm laser (1 W cm^−2^, 5 min). After further incubation for 12 h, therapeutic effects were assessed using the CCK‐8 assay.

Live/dead cell staining was also used for therapeutic effect analysis. RAW264.7 cells activated by LPS (100 ng mL^−1^) were seeded in 6‐well plates and cultured for 24 h. Then, the cells received different administrations (ICG of 40 µg mL^−1^, MTX of 50 µg mL^−1^, mZMI@HA of 160 µg mL^−1^ with the same MTX concentration of 50 µg mL^−1^ and ICG of 40 µg mL^−1^). Then, the cell processing steps were consistent with those mentioned above. For cytokine analyses, the cell culture supernatants were collected after receiving different treatments (ICG of 40 µg mL^−1^, MTX of 50 µg mL^−1^, mZMI@HA of 160 µg mL^−1^ with the same MTX concentration of 50 µg mL^−1^ and ICG of 40 µg mL^−1^), and then performed according to the manufacturer's instructions. Subsequently, the treated RAW264.7 cells were stained with calcein‐AM and EthD‐1 for confocal laser microscopy imaging.

### Collagen‐Induced Rheumatoid Arthritis Model

A collagen‐induced RA model was established according to previously reported protocols. Briefly, the emulsion composed of bovine type II collagen and Complete Freund's Adjuvant was emulsified at a volume ratio of 1:1, and DBA/1 mice received intradermal injection of 100 µL of emulsion at 2 cm from the base of the tail, recorded as day 0. For boost immunization on day 21, the mice were treated with an emulsion containing bovine type II collagen and Incomplete Freund's Adjuvant according to the same means. The mouse RA scoring system was used to assess the progression of inflammation, and paw thickness and joint circumference were measured using a Vernier caliper. Three joint types, the interphalangeal joint, metacarpophalangeal joint, and carpal and tarsal joint, were observed for scoring as follows: normal (Score 0); one joint type had redness and swelling (Score 1); two joint types had redness and swelling (Score 2); all three joint types had redness and swelling (Score 3); maximal redness and swelling of the entire paw led to the disappearance of the anatomical definition (Score 4).

### In Vivo Persistent Luminescence Imaging

To evaluate the targeting ability of mZMI@HA in vivo, normal and RA mouse models were intravenously injected with 200 µL of mZMI@HA (3 mg mL^−1^). Under 2% gas anesthesia, the mice PersL images were recorded by the luminescent in vivo imaging system (IVIS Lumina II) at different time points (0.5, 2, 4, 8, 12, 24, and 48 h). The major organs (heart, liver, spleen, lung, and kidney) and the right hind paws were dissected at 48 h for further PersL imaging analyses. All exposure time was 180 s. The mice fluorescence imaging was recorded using a fluorescence in vivo imaging system (Series III 900/1700). The power of 808‐nm laser was 5 W, exposure time was 50 s.

To study the biodistribution of mZMI@HA, normal DBA/1 mice (*n* = 5) were intravenously injected with 200 µL of mZMI@HA (3 mg mL^−1^). The major organs (heart, liver, spleen, lung, and kidney) were dissected at different time points (6 h, 24 h, 48 h, 72 h, and 7 day) for further PersL imaging analyses (exposure time: 180 s).

### In Vivo Image‐Guided Precision Treatment and Therapeutic Evaluation

After the booster immunization, RA model mice were randomly divided into five groups (*n* = 5): model (G2), PBS with laser (G3), ICG with laser (G4), MTX (G5), and mZMI@HA with laser (G6), in addition to the normal mice, which were treated as G1 with no administration. On days 32, 36, 40, 44, and 48, 200 µL of PBS, MTX, ICG, and mZMI@HA were administered via intravenous injection. After 4 h of administration, the mice in the laser treatment groups were treated with NIR laser irradiation (808 nm, 1 W cm^−2^, 5 min), and photothermal images were recorded using an infrared thermal imager. During treatment, body weight, joint score, joint circumference, and paw thickness were recorded. After the treatment, mouse sera were collected for anti‐inflammatory analyses, and the right hind paws were collected for micro‐CT imaging and histology analyses. For therapeutic evaluation, the mice in all groups received an intravenous injection of 200 µL of mZMI@HA (3 mg mL^−1^), and PersL images were recorded using the PersL imaging system at 4 h post‐injection.

## Conflict of Interest

The authors declare no conflict of interest.

## Supporting information

Supporting Information

## Data Availability

Research data are not shared.

## References

[1] a) M. M. Zaiss , H.‐J. J. Wu , D. Mauro , G. Schet , F. Ciccia , Nat. Rev. Rheumatol. 2021, 17, 224; 33674813 10.1038/s41584-021-00585-3

[2] a) G. S. Firestein , Nature 2003, 423, 356; 12748655 10.1038/nature01661

[3] a) P. Stéphanie , W. René , T. Lahoutte , P. Matthys , Arthritis Res. Ther. 2014, 16, 208; 25099015 10.1186/ar4542 PMC4061725

[4] a) Y. Yuan , Z. Feng , S. L. Li , Z. M. Huang , Y. P. Wan , C. Cao , S. Lin , L. Wu , J. Zhou , L. S. Liao , J. Qian , C. S. Lee , Adv. Mater. 2022, 34, 2201263; 10.1002/adma.202201263 35307885

[5] a) S. J. Zhu , R. Tian , A. L. Antaris , X. Y. Chen , H. Dai , Adv. Mater. 2019, 31, 1900321; 10.1002/adma.201900321 PMC6555689 31025403

[6] a) J. Chen , J. Qi , C. Chen , J. Chen , L. Liu , R. Gao , T. Zhang , L. Song , D. Ding , P. Zhang , C. Liu , Adv. Mater. 2020, 32, 2003399; 10.1002/adma.202003399 32743864

[7] H. H. Wu , Y. N. He , H. Wu , M. J. Zhou , Z. L. Xu , R. Xiong , F. Yan , H. Liu , Theranostics 2020, 10, 10092. 32929336 10.7150/thno.44865 PMC7481417

[8] S. Y. Zhang , L. L. Ning , Z. H. Song , X. Y. Zhao , F. Guan , X. F. Yang , J. Zhang , Anal. Chem. 2022, 94, 5805. 35380780 10.1021/acs.analchem.1c05184

[9] I. Kalashnikova , S.‐J. Chung , M. Nafiujjaman , M. L. Hill , M. E. Siziba , C. H. Contag , T. Kim , Theranostics 2020, 10, 11863. 33204316 10.7150/thno.49069 PMC7667692

[10] a) N. Liu , X. Chen , X. Sun , X. Sun , J. Shi , J. Nanobiotechnol. 2021, 19, 113; 10.1186/s12951-021-00862-z PMC8056701 33879169

[11] a) Z. Li , Q. Wang , Y. Wang , Q. Ma , J. Wang , Z. Li , Y. Li , X. Lv , W. Wei , L. Chen , Q. Yuan , Nano Res. 2018, 11, 6167;

[12] a) D. Ding , Y. Feng , R. Qin , S. Li , L. Chen , J. Jing , C. Zhang , W. Sun , Y. Li , X. Chen , H. Chen , Theranostics 2021, 11, 7439; 34158859 10.7150/thno.62437 PMC8210605

[13] a) Y. Feng , L. C. Zhang , R. Liu , Y. Lv , Biosens. Bioelectron. 2019, 144, 111671; 31513961 10.1016/j.bios.2019.111671

[14] B. Yang , S. Zhou , J. Zeng , L. Zhang , R. Zhang , K. Liang , L. Xie , B. Shao , S. Song , G. Huang , D. Zhao , P. Chen , B. Kong , Nano Res. 2020, 13, 1013.

[15] J. Shi , X. Sun , S. Zheng , L. Song , F. Zhang , Y. Zhang , H. Zhang , M. Hong , ACS Appl. Bio Mater. 2020, 3, 5995. 10.1021/acsabm.0c00644 35021828

[16] a) X. Huang , X. Wei , Y. Zeng , L. Jing , H. Ning , X. Sun , Y. Li , D. Li , Y. Yi , M. Gao , Nanoscale 2021, 13, 8514; 33908437 10.1039/d0nr08267h

[17] a) L. Wu , J. Hu , Q. Zou , Y. Lin , D. Huang , D. Chen , H. Lu , H. Zhu , Nanoscale 2020, 12, 14180; 32602515 10.1039/d0nr03269g

[18] K. Chen , C. Chang , Z. Liu , Y. Zhou , Q. Xu , C. Li , Z. Huang , H. Xu , P. Xu , B. Lu , Colloids Surf., B 2020, 194, 111166. 10.1016/j.colsurfb.2020.111166 32521461

[19] a) J. Fert‐Bober , E. Darrah , F. Andrade , Immunol. Rev. 2020, 294, 133; 31876028 10.1111/imr.12834 PMC8061312

[20] a) Y. Zhu , T. Zhao , M. Liu , S. Wang , S. Liu , Y. Yang , Y. Yang , Y. Nan , Q. Huang , K. Ai , Nano Today 2022, 42, 101358;

[21] a) A. Gadeval , S. Chaudhari , S. P. Bollampally , S. Polaka , D. Kalyane , P. Sengupta , K. Kalia , R. K. Tekade , Drug Discovery Today 2021, 26, 2315; 33962037 10.1016/j.drudis.2021.04.026

[22] L. Liu , F. Hu , H. Wang , X. Wu , A. S. Eltahan , S. Stanford , N. Bottini , H. Xiao , M. Bottini , W. Guo , X. J. Liang , ACS Nano 2019, 13, 5036. 30978282 10.1021/acsnano.9b01710

[23] a) J. Kim , H. Y. Kim , S. Y. Song , S. H. Go , H. S. Sohn , S. Baik , M. Soh , K. Kim , D. Kim , H. C. Kim , N. Lee , B. S. Kim , T. Hyeon , ACS Nano 2019, 13, 3206; 30830763 10.1021/acsnano.8b08785

