# Supplementary material for: Dual‐Triggered Near‐Infrared Persistent Luminescence Nanoprobe for Autofluorescence‐Free Imaging‐Guided Precise Therapy of Rheumatoid Arthritis
Source: Adv Sci (Weinh). 2022 Dec 3;10(4):2205320. doi: 10.1002/advs.202205320 (PMC9896051; doi:10.1002/advs.202205320)
Supplement: Supplementary file 1 — Supporting Information [file ADVS-10-2205320-s001.pdf]

Supporting Information

Dual-Triggered Near-Infrared Persistent Luminescence Nanoprobe for Autofluorescence-Free Imaging-Guided Precise Therapy of Rheumatoid Arthritis

Ruoping Wang, Junpeng Shi, Qian Zhang, Qiang Peng, Xia Sun, Liang Song, and Yun Zhang**

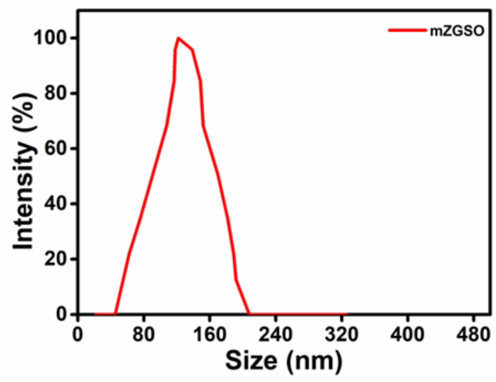

Figure S1. DLS analysis of mZGSO.

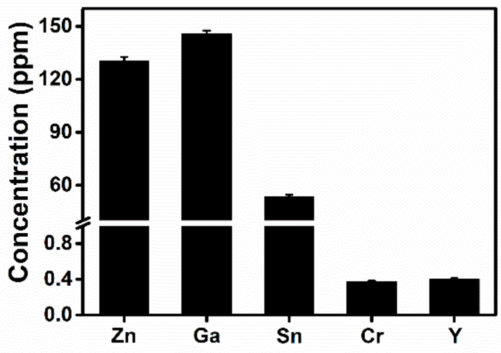

Figure S2. Quantitative analyses the concentrations of metal elements (Zn, Ga, Sn, Cr, Y) in mZGSO.

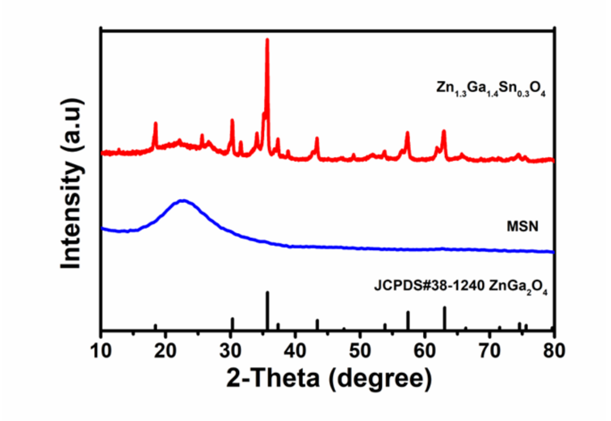

Figure S3. XRD patterns of mZGSO and MSN.

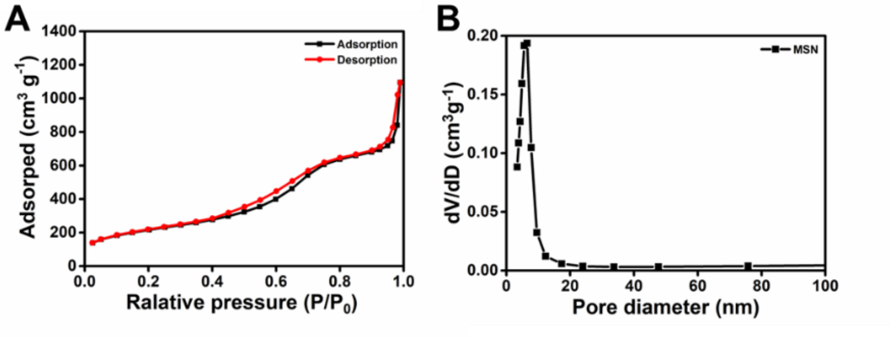

Figure S4. A) N_2 adsorption/desorption isotherms of MSN. B) Pore size distribution of MSN.

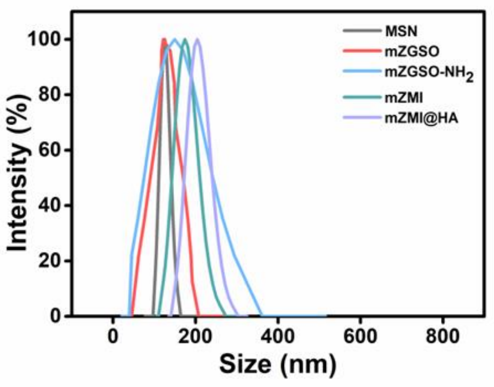

Figure S5. DLS analyses of the prepared nanoparticles**.**

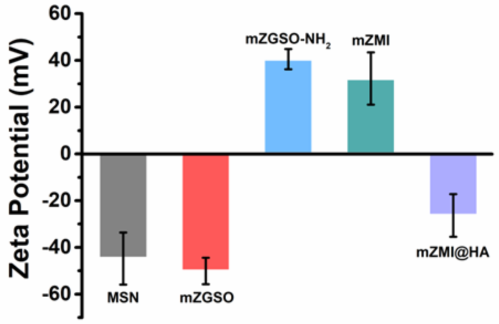

Figure S6. Zeta potential of the prepared nanoparticles**.**

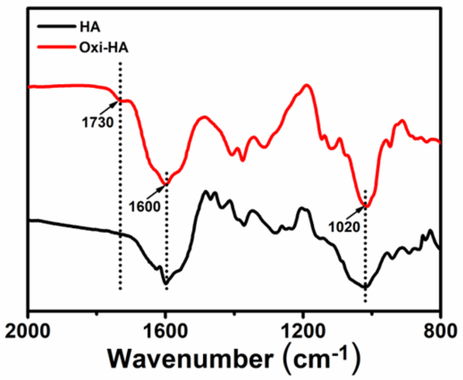

Figure S7. FTIR spectrums of HA and oxi-HA.

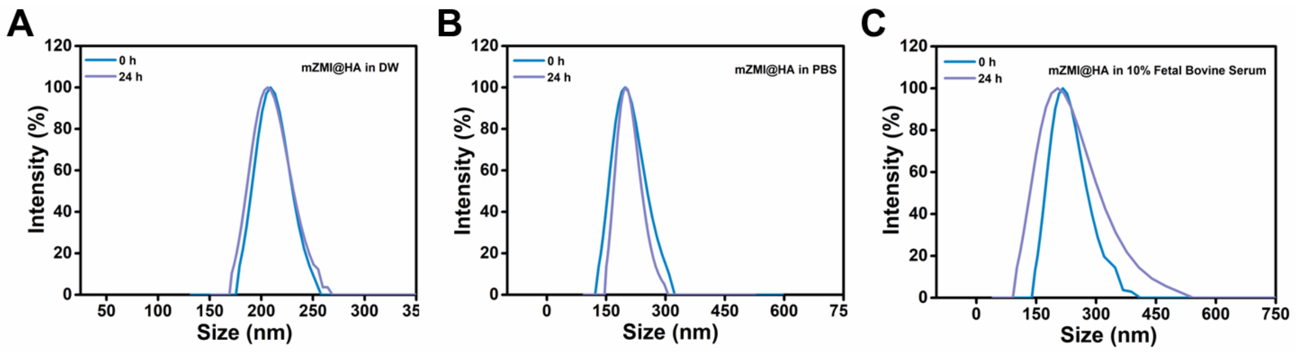

Figure S8. Stability analyses of mZMI@HA. A) DLS analysis of mZMI@HA in deionized water (DW). B) DLS analysis of mZMI@HA in pH 7.4 PBS. C) DLS analysis of mZMI@HA in 10% fetal bovine serum.

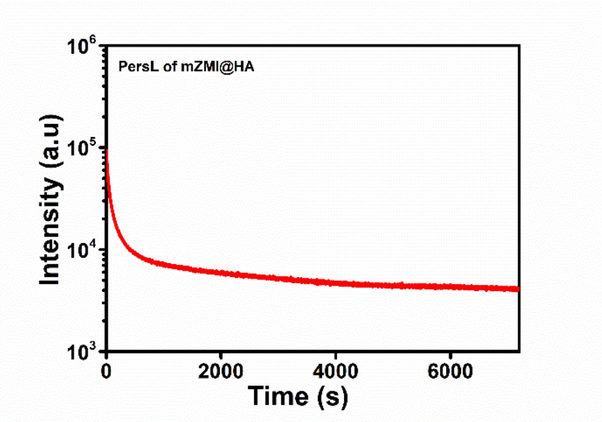

Figure S9. PersL decay curves of mZMI@HA upon 659-nm LED light irradiation for 5 min (monitored at 700 nm).

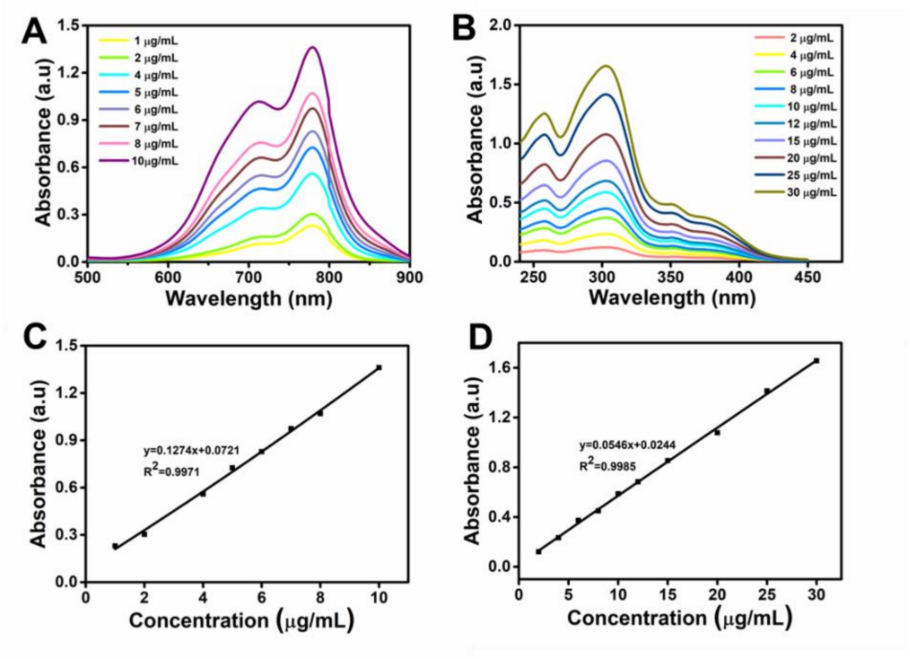

Figure S10. A) UV/vis absorption spectra of ICG. B) UV/vis absorption spectra of MTX. C) Calibration curve of ICG. D) Calibration curve of ICG.

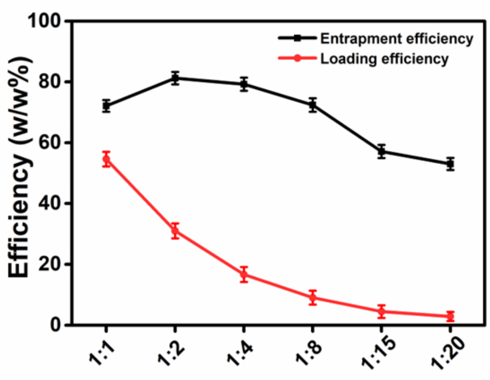

Figure S11. Entrapment efficiency and loading efficiency of mZMI**.**

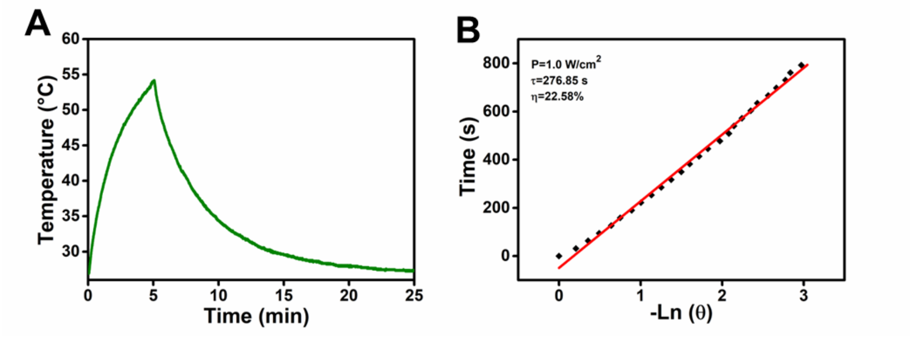

Figure S12. Photothermal effect of mZMI@HA upon 808-nm irradiation (1.0 W cm^{-2}, 5 min). A) The heating and cooling stage of mZMI@HA. B) The time constant of mZMI@HA, heat transfer mechanism from the system as determined by using the linear time data from the cooling period.

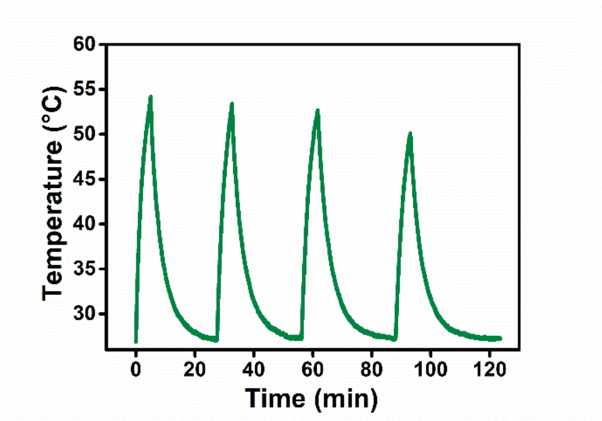

Figure S13. Photothermal stability performance of mZMI@HA upon 808-nm irradiation (1.0 W cm^{-2}, 5 min) for four on-and-off cycles.

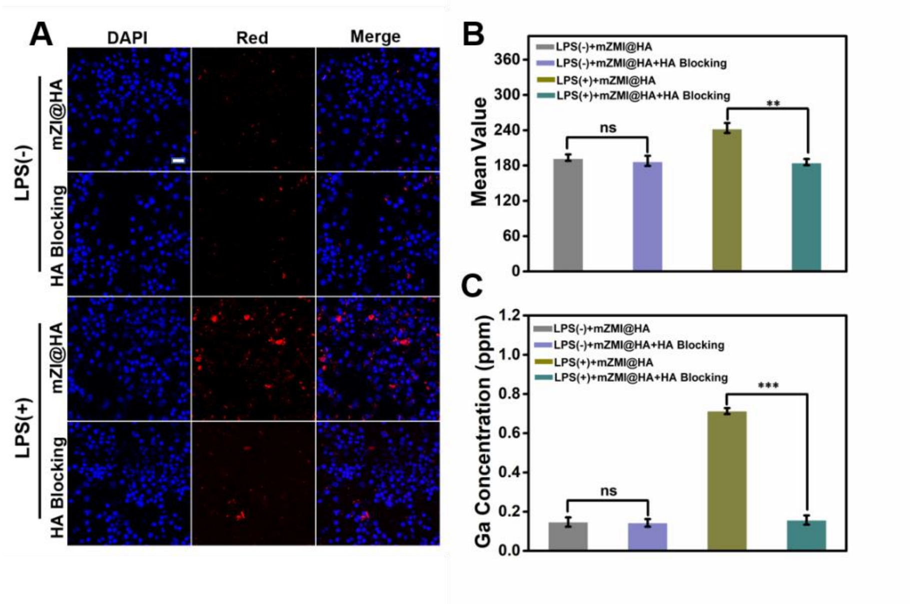

Figure S14. Validation of the CD44 overexpression on RAW264.7 cells. A) Cell images of RAW264.7 cells (administration of mZI@HA) observed under confocal laser scanning microscopy (scale bar: 25 μm). B) Statistical analyses of the cell images. C) Quantitative analyses of cellular uptake behaviors of mZI@HA by ICP-OES. $^{***}P < 0.001$, $^{**}P < 0.01$, *ns* refers to no statistical difference.

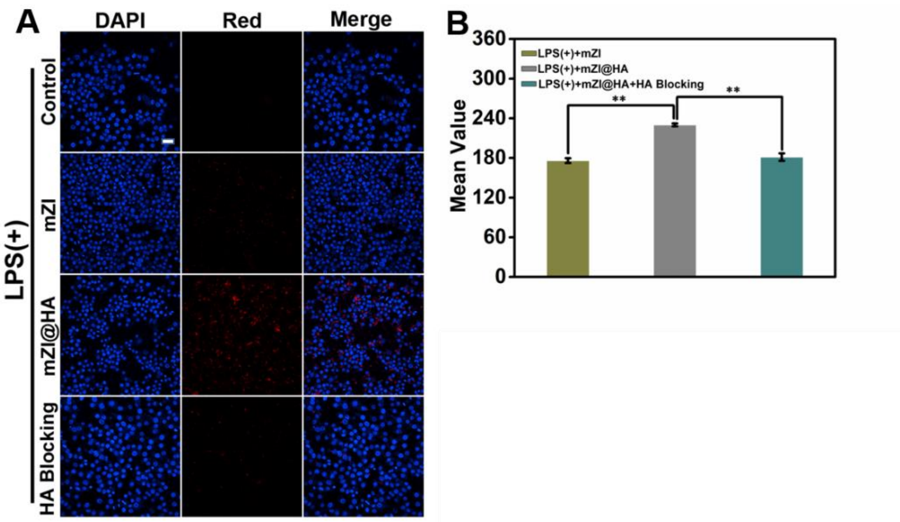

Figure S15. Targeting capability of mZI@HA. A) Cell images of LPS-stimulated RAW264.7 cells (administration of different treatments) observed under confocal laser scanning microscopy (scale bar: 25 μm). B) Statistical analyses of the cell images. $**P < 0.01$.

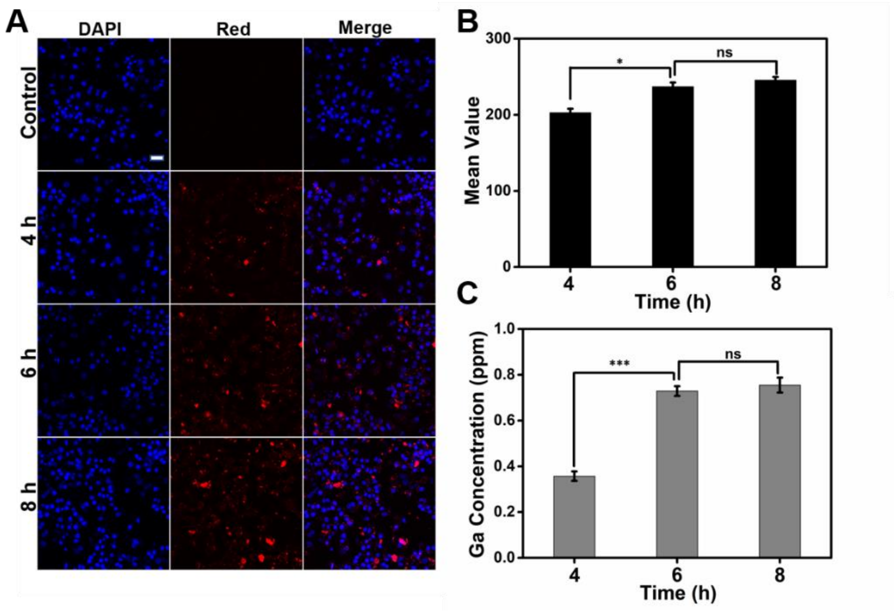

Figure S16. Incubation time dependent cellular uptake behaviors of mZI@HA. A) Cell images of LPS-stimulated RAW264.7 cells observed under confocal laser scanning microscopy (scale bar: 25 μm). B) Statistical analyses of the cell images. C) Quantitative analyses of cellular uptake behaviors of mZI@HA by ICP-OES. $^{***}P < 0.001$, $^{*}P < 0.05$, ns refers to no statistical difference.

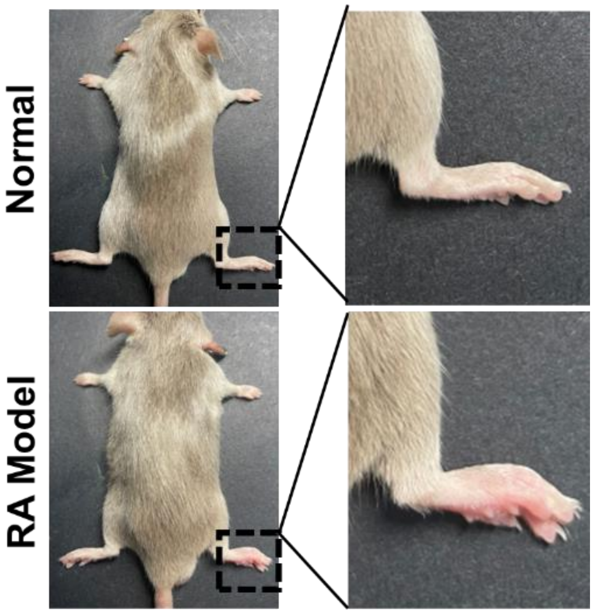

Figure S17. Images of normal mice and RA model.

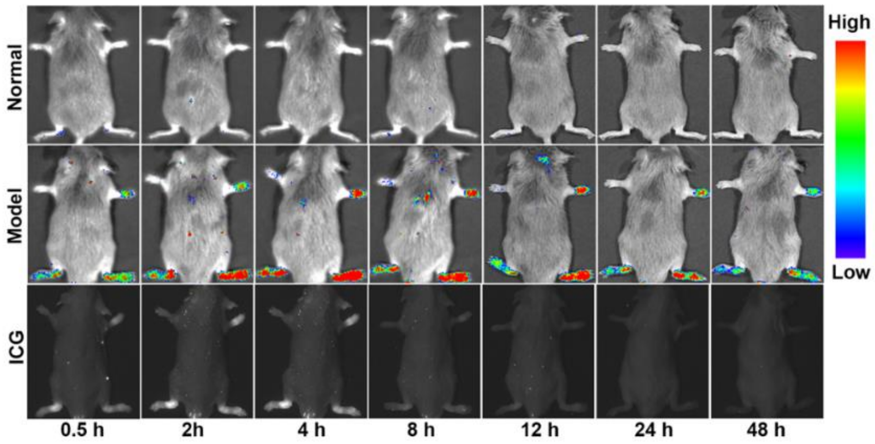

Figure S18. PersL and fluorescence imaging of mice treated with mZMI@HA at different time points.

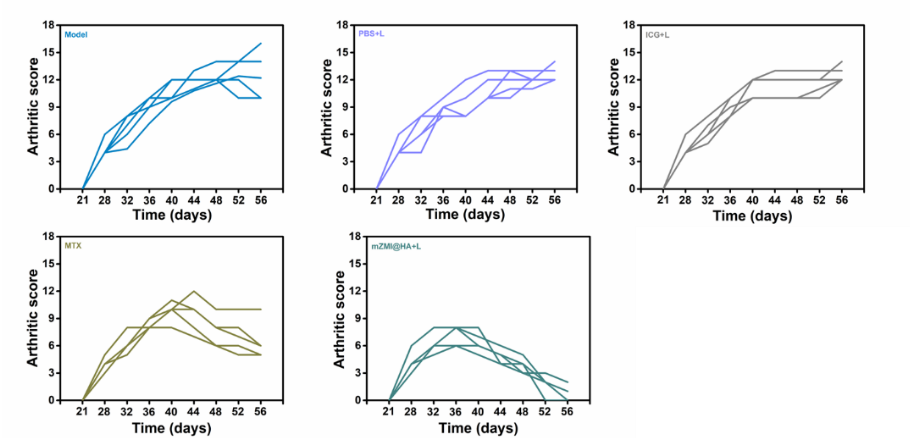

Figure S19. The arthritis score changes of typical mice receiving different treatments (*n*=5).

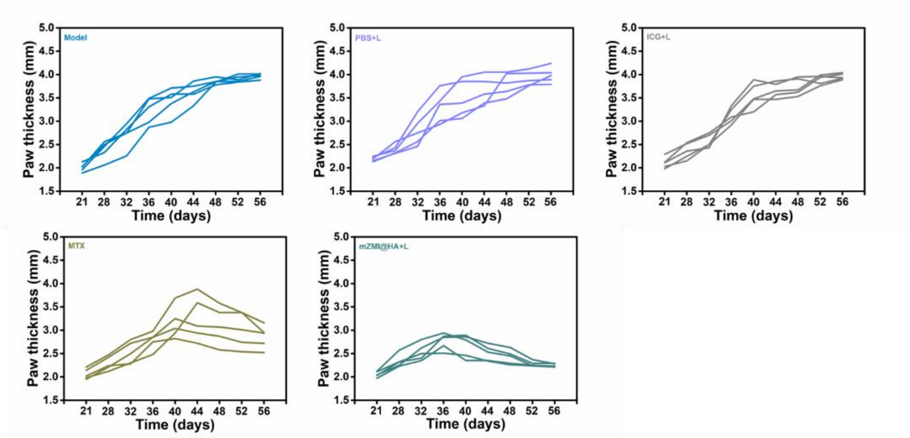

Figure S20. The paw thickness changes of typical mice receiving different treatments (*n*=5).

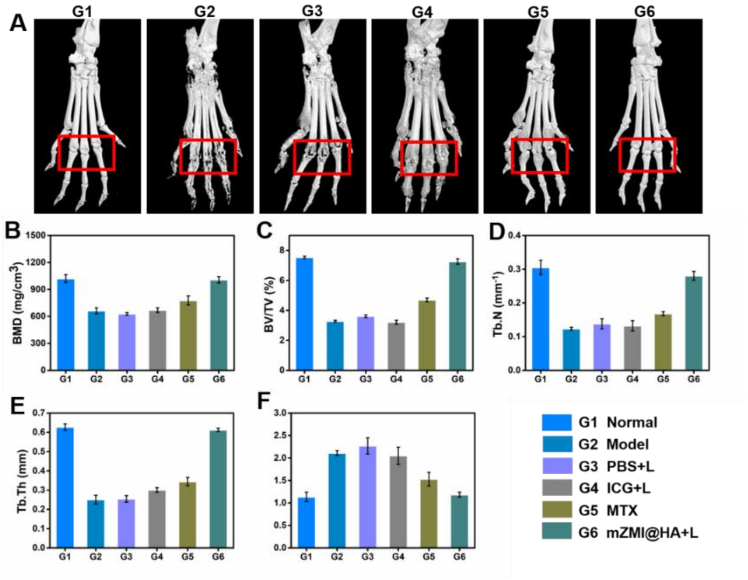

Figure S21. A) Micro-CT images of the right hind forepaws on day 56. B, C) Quantitative micro-CT analysis of BMD and BV/TV in finger joints. D-F) Quantitative micro-CT analyses of trabecular number (Tb.N), trabecular bone thickness (Tb.Th), and trabecular separation (Tb.Sp) in finger joints.

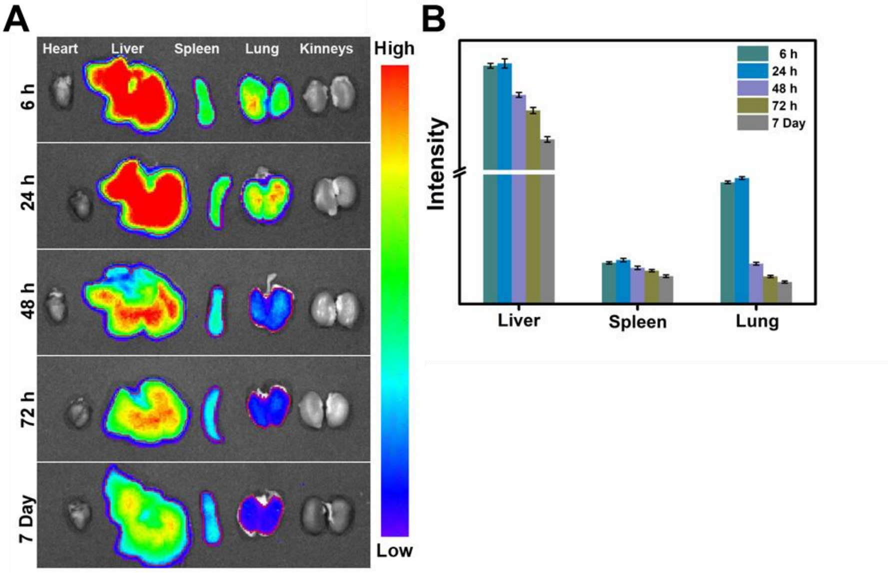

Figure S22. Biodistribution of mZMI@HA in major organs after intravenous administration at different time intervals (*n*=3). A) PersL imaging of major organs. B) Statistical analyses of the PersL images.

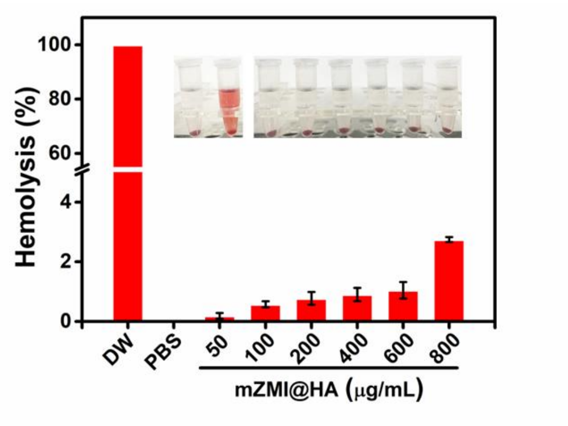

Figure S23. The hemolysis test of mZMI@HA with various concentrations at 37 °C for 4 h.

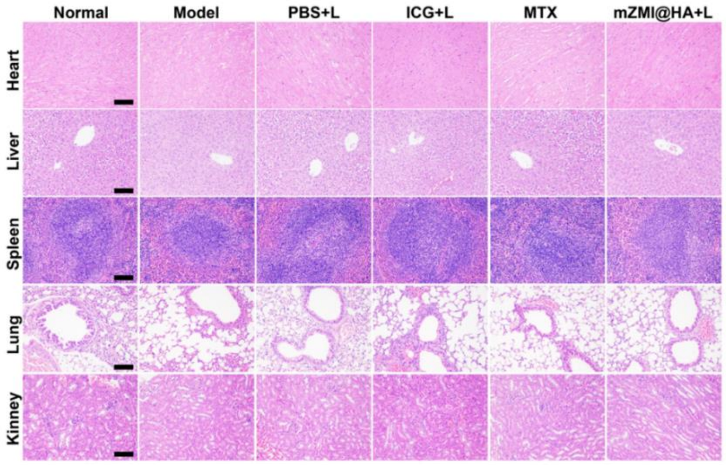

Figure S24. H&E images of major organs.

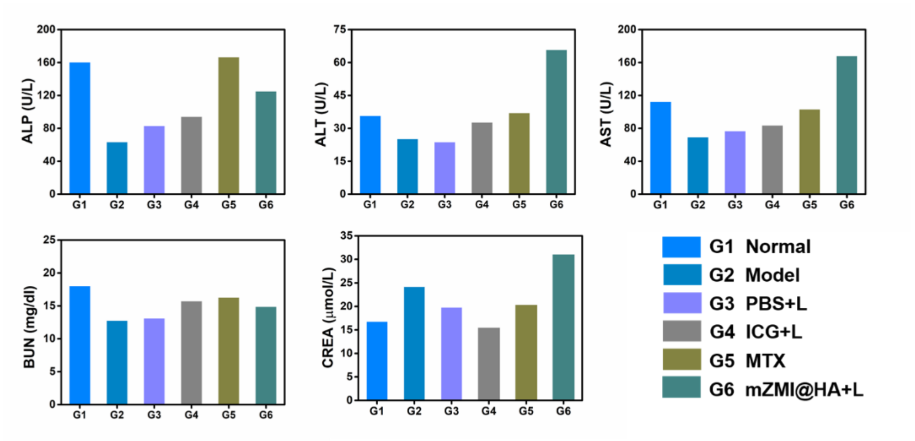

Figure S25. Blood biochemical tests of mice receiving different treatments.
